# Adaptiveness of dark personalities: psychopathy and sadism have opposite associations with fertility

**DOI:** 10.3389/fpsyg.2025.1644767

**Published:** 2025-09-09

**Authors:** Janko Međedović, Ivana Hromatko

**Affiliations:** ^1^Institute of Criminological and Sociological Research, Belgrade, Serbia; ^2^Department of Psychology, Faculty of Humanities and Social Sciences, University of Zagreb, Zagreb, Croatia

**Keywords:** dark tetrad, psychopathy, sadism, fertility, natural selection

## Abstract

Analyzing the links between behavioral traits and fertility is a pivotal step toward understanding their current adaptiveness and the potential regimes of natural selection acting upon them. In this study, we examined associations between the Dark Tetrad traits—psychopathy, narcissism, Machiavellianism, and sadism—and two fertility indicators: number of children and age of first reproduction, in a representative probabilistic sample of Croatian citizens (*N* = 690). Regression analyses showed that psychopathy positively predicted the number of children and negatively predicted age of first reproduction; the opposite pattern was observed for sadism. Mediation analysis further revealed that age of first reproduction mediated the relationship between sadism and number of children, while the mediation effect for psychopathy was marginally significant. These findings add to existing evidence suggesting that psychopathy may confer adaptive advantages in terms of evolutionary fitness, potentially supporting positive directional selection for this trait. In contrast, sadism may be maladaptive in an evolutionary context. Additionally, the results contribute to the ongoing debate regarding the potential redundancy of psychopathy and sadism as behavioral dispositions, indicating that such a conclusion may be premature.

## 1 Introduction

### 1.1 Dark tetrad traits: analyzing fertility as a window in their behavioral evolution

Human behavioral scientists have devoted considerable attention over the past several decades to describing malevolent, malicious, immoral, and socially destructive behavioral dispositions. The first formal taxonomy of such traits was introduced as the *Dark Triad* (Paulhus and Williams, 2002; Furnham et al., 2013), comprising: psychopathy (characterized by callousness, lack of empathy, and guiltlessness), Machiavellianism (marked by manipulativeness and interpersonal ruthlessness aimed at achieving personal goals), and narcissism (defined by self-centeredness, arrogance, and a sense of entitlement).

Later, researchers proposed that this taxonomy was incomplete without accounting for the personality trait involving pleasure derived from harming others or witnessing their distress—referred to as *everyday sadism* (Chabrol et al., 2009; Paulhus, 2014). This addition gave rise to the expanded *Dark Tetrad* (hereafter: DT). A current debate within DT research concerns the potential redundancy between psychopathy and sadism, as their nomological networks appear highly overlapping—raising the possibility that they may reflect distinct facets of a single broader underlying construct (Blötner and Mokros, 2023; Blötner et al., 2024). Unsurprisingly, given their content, DT traits have been empirically linked to immoral, antisocial, aggressive, and criminal behaviors (e.g., Chabrol et al., 2009; Paulhus, 2014; Blötner et al., 2021).

DT traits are also known to be moderately heritable (Vernon et al., 2008), which makes them viable candidates for natural selection. (To date, no data are available on the heritability of sadism. However, there is no reason to assume that its genetic architecture differs significantly from that of other dark traits.) A fundamental method for examination of the possibility for phenotypic selection is by analyzing how traits relate to fertility—an essential component of evolutionary fitness. So, how do DT traits relate to fertility-related outcomes?

Current evidence suggests that psychopathy may be at least partly adaptive, showing positive associations with fertility (Brazil and Volk, 2025; Ene et al., 2022; Međedović, 2023). This is particularly true for its affective components (e.g., low empathy, blunted negative emotions) and interpersonal style (e.g., manipulativeness, deceitfulness). Findings for narcissism are more mixed—some studies report positive associations (Carter et al., 2018; Međedović, 2025a), some find null effects (Međedović, 2025b), and yet others suggest that narrow facets of narcissism may even show divergent relationships with fitness (Međedović and Jovanov, 2025). Research on Machiavellianism and sadism remains sparse, and sadism may prove maladaptive given its nature as an extreme and dysregulated form of aggressiveness.

Several hypotheses have emerged regarding the evolutionary underpinnings of DT traits. One suggests that they represent behavioral phenotypes aligned with a *fast life-history strategy* (Réale et al., 2010), which favors earlier reproduction and greater reproductive output (Jonason et al., 2010). Another posits that DT traits—especially psychopathy—may be more adaptive in males than in females (Međedović, 2023). A third theoretical proposition argues that DT traits might yield greater fitness in densely populated urban environments (and conversely—lower fitness in smaller and rural settlements), although the existing data supported this hypothesis only for narcissism (Međedović, 2025b). Altogether, analyzing the links between DT traits and fertility offers valuable insights into current selection regimes and the possible evolutionary trajectories of malevolent and immoral personality dispositions.

### 1.2 Goals of the present research

The primary aim of the present study is to investigate the relationships between Dark Tetrad (DT) traits, age of first reproduction, and number of children in a diverse and heterogeneous general population sample. This research contributes to literature in several important ways. First, empirical studies examining the associations between DT traits and fertility are still relatively scarce. Some existing studies have focused only on the Dark Triad, excluding sadism altogether (e.g., Carter et al., 2018), while others have been conducted within highly specific populations, such as incarcerated individuals (Međedović, 2025a). Second, analyses incorporating age of first reproduction—a key indicator of pace-of-life syndrome—and its associations with DT traits are virtually nonexistent, to the best of our knowledge. Current findings suggest that psychopathy exhibits the strongest adaptive potential from an evolutionary perspective, followed by narcissism; in contrast, sadism appears to be largely maladaptive with respect to reproductive fitness. We extend this logic to the age of first reproduction: under the fast life-history hypothesis, the adaptive potential of DT traits may manifest through earlier age of first reproduction. Accordingly, these relationships form our central hypotheses. Due to insufficient prior evidence, we do not offer a specific directional hypothesis regarding Machiavellianism; its role is treated as exploratory in this analysis. Additionally, the study tests two further hypotheses: (1) Previous research suggests that DT traits may confer greater adaptive benefits to males than females, which is why we include sex as a potential moderator of the relationship between DT traits and reproductive outcomes; (2) There are also indications that DT traits may be more adaptive in densely populated (urban) environments than in smaller settlements, prompting us to explore settlement size as a second moderator.

## 2 Methods

### 2.1 Procedure

The data used in this study were originally collected as part of a larger research project; however, the current paper is not directly related to that project and is based on secondary analyses of the dataset. The data were collected by the research agency Ipsos using their established online panel survey infrastructure. Participants were drawn from a pre-recruited panel and received compensation for their participation. Ipsos employs stratified quota sampling to ensure that the final sample is demographically representative of the national population in terms of key variables such as age, gender, region, and education. A nationally representative probabilistic sample (*N* = 690) was selected using a two-stage stratified sampling procedure based on region and settlement size. Additional quota controls were applied to ensure balanced representation by age and education level. The survey was administered online, during March 2025. All participants provided informed consent prior to participation. Participants first responded to demographic items (age, gender, education, socioeconomic status, employment status, number of children, and age at first reproduction), followed by the Short Dark Tetrad scale (Paulhus et al., 2021a; Paulhus et al., 2021b) and additional items not relevant to the present study (e.g., questions on digital media use, trust in institutions, political attitudes, etc.). There were no missing data.

### 2.2 Instruments

The Short Dark Tetrad scale (Paulhus et al., 2021a; Paulhus et al., 2021b; Croatian translation and adaptation, Wertag et al., 2023) is an instrument consisting of 28 items, with 7 items for each of the four dark personality traits: Machiavellianism, narcissism, psychopathy, and sadism. Agreement with the statements is rated on a 5-point Likert scale, where 1 indicates “strongly disagree” and 5 indicates “strongly agree.” The total score for each dark trait is calculated as the mean value of the seven corresponding items.

### 2.3 Participants

Out of the 690 Croatian citizens who participated in this study, *n* = 348 (50.4%) were women. The average age of participants was *M* = 47.7 years (*SD* = 12.38; range = 21–66). In terms of educational attainment, 2.0% had completed elementary school, 55.4% had graduated from vocational school, 11.7% had completed a gymnasium-type high school, 12.3% held a bachelor’s degree, and 1.9% held a master’s degree or PhD. The participants’ average monthly income was €1,150, which aligns with the national median wage for March 2025 (Croatian Bureau of Statistics). Participants were also diverse in terms of settlement size: 35.9% lived in communities with up to 2,000 residents, 14.9% in settlements with 2,001 to 10,000 residents, 21.4% in towns with 10,001 to 80,000 residents, and 27.7% in cities with more than 80,000 residents. Regarding the key dependent variables, the age at first reproduction ranged from 14 to 50 years (*M* = 27.06; *SD* = 5.64), while the number of children ranged from 0 to 6 (*M* = 1.36; *SD* = 1.14). This demographic profile clearly reflects the probabilistic and stratified nature of the sample, ensuring it is not biased toward younger, more educated, predominantly female participants who are often overrepresented in online studies.

## 3 Results

### 3.1 Descriptive statistics, reliabilities and the correlations between examined measures

Firstly, we report the means, standard deviations, and reliability coefficients (Cronbach’s *α*) for the DT scales. These descriptive statistics, along with Spearman’s correlation coefficients among the analyzed variables, are presented in Table 1. The mean values of Dark Tetrad traits are very similar to those reported in geographically and culturally neighboring countries (Dinić et al., 2024), with fertility and age at first reproduction roughly corresponding at the population level. All DT traits demonstrated acceptable internal consistency, except for Machiavellianism, which showed a somewhat lower alpha value. The DT traits were positively intercorrelated, with associations of moderate magnitude. Regarding fertility outcomes, Machiavellianism, narcissism, and sadism were negatively associated with the number of children, while sadism also showed a positive correlation with age of first reproduction. As expected, the two fertility measures—number of children and age of first reproduction—were negatively correlated. Sex differences in DT and fertility measures, together with correlations between age, education, settlement size, SES, DT, and fertility measures are shown in Supplementary material.

**Table 1:** Descriptive statistics, reliabilities, and the correlations between the examined variables.

Variables	M(SD)	α	1	2	3	4	5
1. Machiavellianism	3.16 (0.62)	0.68					
2. Narcissism	2.55 (0.65)	0.75	0.41**				
3. Psychopathy	1.85 (0.63)	0.76	0.36**	0.52**			
4. Sadism	1.73 (0.62)	0.73	0.37**	0.41**	0.55**		
5. Number of children	1.36 (1.14)		−0.12**	−0.09*	−0.01	−0.18**	
6. Age of first reproduction	27.10 (5.64)		0.07	0.07	−0.01	0.13**	−0.31**

*p <0.05; **p < 0.01.

### 3.2 Predicting the number of children and age of first reproduction: the regression models

Next, we fitted two regression models to examine the predictive effects of DT traits and sociodemographic variables on fertility outcomes. The first model predicted the number of children using Poisson regression, while the second employed linear regression to explain variation in age of first reproduction. Both models included participants’ sex, age, education, socioeconomic status (SES), and settlement size as covariates. Results are presented in Table 2. For the number of children, significant positive predictors included psychopathy, SES, and participants’ age. In contrast, lower sadism, smaller settlement size, lower educational level, and female sex also made significant contributions to the model. Regarding the age of first reproduction, the predictors were lower psychopathy and male sex, along with higher sadism and higher educational attainment—all associated with later reproduction. No significant interactions were found between DT traits and participants’ sex, nor between DT traits and settlement size in predicting either fertility outcome.

**Table 2:** Prediction of the fertility measures by the dark tetrad traits.

Variables	Number of children	Age of first reproduction
Machiavellianism	−0.06 (0.06)	0.01 (0.43)
Narcissism	−0.05 (0.07)	0.04 (0.47)
Psychopathy	0.17 (0.08)*	−0.12 (0.54)*
Sadism	−0.18 (0.08)**	0.12 (0.54)*
Settlement size	−0.13 (0.03)**	0.08 (0.20)
Sex	−0.22 (0.07)**	−0.27 (0.49)**
Age	0.03 (0.00)**	−0.01 (0.02)
Education	−0.06 (0.03)**	0.25 (0.18)**
SES	0.04 (0.02)**	0.02 (0.13)
Model fit	*AIC* = 1907.4	*F* = 12.54**
R^2^	0.30	0.44

Unstandardized coefficients with standard errors in parentheses are provided for the Number of children as a criterion measure; standardized coefficients with standard errors in parentheses are provided for the Age of first reproduction as a criterion measure; Poison’s regression do not report F square and its significance, only the AIC parameter; *p < 0.05; **p < 0.01.

### 3.3 Does the age of first reproduction mediate the link between psychopathy, sadism, and number of children?

Finally, we examined whether age of first reproduction mediates the relationship between DT traits and number of children. As a first step, we modeled DT traits as latent variables, with questionnaire items serving as observed indicators. Participants’ sex, age, education, socioeconomic status (SES), and settlement size were included as control variables to ensure a stringent and reliable analysis. However, this model did not achieve acceptable fit indices [χ^2^(146) = 420.11, *p* > 0.05; *CFI* = 0.864; *TLI* = 0.841; *RMSEA* = 0.063; *SRMR* = 0.054], likely due to the large number of observed indicators for the DT constructs, and the sample size that might not be high enough for a reliable estimation of the model with latent variables.

**Figure 1:**
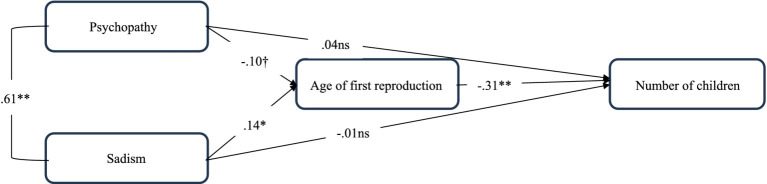
Mediating role of age of first reproduction in the links between psychopathy, sadism, and number of children. Two-arrowed path represent correlation; one-arrowed paths represent regressions; ns, not significant; ^†^*p* = 0.07; *p < 0.05; **p < 0.01.

Consequently, we conducted a path analysis in which all variables were treated as observed, using robust maximum likelihood estimation. In this model, narcissism and Machiavellianism were not significantly associated with either of the fertility outcomes and were therefore excluded from the final analysis. In the final model (see Figure 1), the age of first reproduction negatively predicted the number of children. Psychopathy was marginally negatively associated with the age of first reproduction, whereas sadism showed a significant positive association. The model demonstrated good fit: χ^2^(10) = 10.448, *p* > 0.05; *CFI* = 0.999; *TLI* = 0.998; *RMSEA* = 0.010; *SRMR* = 0.029. The indirect effect of sadism on the number of children was statistically significant (*β* = −0.04, *p* = 0.016), while the indirect effect of psychopathy was marginally significant (*β* = 0.03, *p* = 0.069). These results suggest that the age of first reproduction fully mediates the relationships between psychopathy, sadism, and number of children that were previously identified in the regression models.

## 4 Discussion

In the present study, we examined the associations between Dark Tetrad (DT) traits and two key indicators of fertility—number of children and age of first reproduction. These associations were used to infer the potential evolutionary adaptiveness of dark personality traits, given that fertility is a central component of evolutionary fitness (i.e., the process by which gene allele frequencies shift across generations). In terms of sex, age, education, socioeconomic status, number of children, and age of first reproduction—our sample closely mirrors that of the general Croatian population. This alignment allows for extrapolation of our findings to broader population-level processes of natural selection acting on DT traits. Our primary findings, derived from multivariate models, indicate that psychopathy and sadism show opposite relationships with fertility outcomes: psychopathy is associated with higher reproductive success, while sadism is linked to lower fertility. These patterns suggest that psychopathy may be subject to positive directional selection, whereas sadism could be under negative selection pressure within this population. Contrary to our hypotheses, we did not observe significant interactions between DT traits, sex, or settlement size in predicting fertility outcomes.

### 4.1 Psychopathy and sadism may be targeted by opposite processes of natural selection

Some discrepancies emerged between our bivariate and multivariate analyses; however, we focus our interpretation primarily on the multivariate models, as they account for key covariates—namely sex, age, education, socioeconomic status, and settlement size—all of which were significant predictors in the regression models, at least for number of children. The positive association between psychopathy and number of offspring aligns with most existing findings (Brazil and Volk, 2025; Ene et al., 2022; Međedović, 2023). Nonetheless, it is important to note that some studies did not observe such a relationship (Carter et al., 2018; Međedović, 2025a). The discrepancies in findings across studies may be attributed to the use of different DT inventories and substantial variation in the populations examined (e.g., general population vs. incarcerated individuals). Researchers have proposed that psychopathy may be evolutionarily adaptive, particularly through its interpersonal and affective dimensions: traits such as manipulativeness and deceitfulness may confer interpersonal advantages, including enhanced reproductive success, while reduced fear, guilt, and anxiety could promote stress resilience (Glenn et al., 2011). Moreover, previous work has suggested that psychopathy may reflect a behavioral phenotype consistent with a fast pace-of-life syndrome (Jonason et al., 2010), contributing to fitness via earlier age of first reproduction (Réale et al., 2010). However, to the best of our knowledge, no prior study has empirically demonstrated a direct link between psychopathy and the age of first reproduction. Thus, our findings provide the first indication that psychopathy may indeed function as a personality marker of a faster pace-of-life, with its reproductive advantage potentially mediated by earlier reproductive timing.

In contrast to psychopathy, our findings for sadism indicate the opposite pattern. The only existing study examining sadism and fertility yielded inconclusive results, reporting non-significant associations with number of children and some indications of earlier age of first reproduction (Pavlović and Međedović, 2024). In the present study, however, the findings are more clear-cut: individuals exhibiting higher levels of sadism tend to have fewer children, a pattern that appears to be driven by delayed onset of reproduction. While traits such as aggression and anger may be adaptive in certain contexts—such as self-defense or achieving social status through dominance—sadism reflects a hypertrophied and excessively modulated form of aggression, one that serves no evident functional purpose beyond the experience of pleasure in inflicting harm. Given this, it is unsurprising that sadism has been consistently linked to a range of psychopathological outcomes (Myers et al., 2006; Paulhus et al., 2021a; Paulhus et al., 2021b). Moreover, it is well established that more severe forms of psychopathology tend to reduce evolutionary fitness (Keller and Miller, 2006). Taken together, our findings support the interpretation of sadism as a maladaptive, destructive form of aggression that, in the current population, may be subject to negative selection pressures.

It is important to emphasize that the data from the present study provide more conclusive evidence for sadism than for psychopathy. Despite fully significant regression coefficients, psychopathy showed only marginally significant association with the age of first reproduction in the structural model and nonsignificant zero-order associations with the criteria measures. On the other hand, significant associations involving sadism emerged consistently across all three analytical approaches; this finding has never been reported so far and this is the major contribution of the present research. Despite this difference in robustness, the findings make a valuable contribution to the ongoing debate concerning the potential redundancy or equivalence of psychopathy and sadism. Specifically, previous research has suggested that psychopathy and sadism share highly similar nomological networks, raising the possibility that they may not represent distinct psychological constructs but rather reflect different manifestations of a single underlying personality trait (Blötner and Mokros, 2023; Blötner et al., 2024). However, our findings challenge this view by demonstrating that psychopathy and sadism show opposing relationships with one of the most fundamental evolutionary biological outcomes—fertility. This contrast suggests that the two traits may differ significantly in their adaptive potential and evolutionary trajectories. Accordingly, the redundancy hypothesis warrants further empirical scrutiny, and the present results underscore the theoretical and practical value of assessing psychopathy and sadism as distinct dimensions of personality.

### 4.2 Explanations of hypothesized effects that we did not obtain

We would also like to address several associations previously reported in the literature that were not observed in the current study. Specifically, although we found negative bivariate correlations between Machiavellianism, narcissism, and number of children, these effects did not remain significant in our multivariate models. Prior research has yielded mixed findings regarding the relationship between narcissism and fertility, including negative (Međedović and Jovanov, 2025), null (Međedović, 2025b), and positive associations (Carter et al., 2018; Međedović, 2025a). This variability suggests that the link between narcissism and reproductive outcomes may depend on specific facets of the trait, which can have opposing associations with fertility (Međedović and Jovanov, 2025). Therefore, future research would benefit from a more fine-grained approach that distinguishes among narcissism’s subcomponents—particularly grandiose vs. vulnerable narcissism, or narcissistic admiration vs. rivalry. A similar pattern applies to psychopathy. Previous studies have identified moderation effects by sex when examining narrower psychopathy traits (Međedović, 2023), but such effects were not replicated in our analyses. This further supports the need to consider the distinct facets of psychopathy, especially given the divergent associations of manipulative/affective traits and impulsive/disinhibited traits with fertility-related outcomes. Finally, although prior research found that narcissistic individuals tend to have lower fertility in smaller settlements (Međedović, 2025b), we did not observe this effect. This discrepancy may stem from differences in sample size, demographic composition, or broader cultural context. Collectively, these inconsistencies highlight the importance of examining both broad and narrow trait dimensions and ensuring sufficient statistical power in future studies.

### 4.3 Limitations, future directions, and conclusions

Several limitations of the present study should be acknowledged. First, the Dark Tetrad (DT) measures employed were brief and unidimensional, meaning they did not capture the narrower facets of each DT trait. This is a notable limitation, as previous research has demonstrated that specific subdimensions of DT traits may relate differently to fertility outcomes. Although the structure and demographic diversity of our sample represent key strengths, a considerable proportion of participants were still within their reproductive years. While we statistically controlled for age, this nonetheless limits the accuracy of the number of children as a definitive indicator of lifetime reproductive success. Furthermore, the study employed a cross-sectional design. Robust evidence of natural selection ideally requires longitudinal data in which psychological traits are assessed prior to the realization of fitness outcomes.

Despite these limitations, the present findings contribute meaningfully to our understanding of the potential adaptiveness of DT traits, their placement within pace-of-life frameworks, and their possible roles in ongoing selection processes. Findings showed negative adaptive potentials of sadism reflected in lower fertility, which are in accordance with largely maladaptive features of this trait in a proximal sense as well; as far as we are aware, this is the first study to report these effects. On the contrary, and in congruence with previous research, psychopathy showed some phenotypic signals of adaptive evolution under positive directional selection although with less conclusiveness compared to the effects obtained for sadism. Future studies could expand on these insights by utilizing more comprehensive DT measures, incorporating ecological conditions in study designs, and examining a broader range of fitness-related outcomes to more fully elucidate the evolutionary trajectories of dark personality traits.

## Data Availability

The raw data supporting the conclusions of this article will be made available by the authors, without undue reservation.
