# Supplementary material for: Adaptiveness of dark personalities: psychopathy and sadism have opposite associations with fertility
Source: Front Psychol. 2025 Sep 9;16:1644767. doi: 10.3389/fpsyg.2025.1644767 (PMC12454322; doi:10.3389/fpsyg.2025.1644767)
Supplement: Supplementary file 1 [file Table_1.DOCX]

**Supplementary material**

**Table S1**

*Sex differences in the Dark Tetrad traits and fertility measures*

|  | M(SD)_males_ | M(SD)_females_ | t |
| --- | --- | --- | --- |
| Machiavellianism | 3.21(0.61) | 3.10(0.62) | 2.40* |
| Narcissism | 2.61(0.62) | 2.49(0.67) | 2.44* |
| Psychopathy | 1.96(0.64) | 1.75(0.60) | 4.35*** |
| Sadism | 1.89(0.68) | 1.58(0.52) | 6.58*** |
| Number of children | 1.17(1.14) | 1.54(1.11) | -4.26*** |
| Age at first reproduciton | 29.04(5.59) | 25.54(5.19) | 7.05*** |

Notes: * - *p* < .05; *** - *p* < .001

**Table S2**

Spearman’s correlations coefficients between, age, education, SES, Settlement size, Dark Tetrad and fertility measures

|  | Age | Education | SES | Settlement size |
| --- | --- | --- | --- | --- |
| Machiavellianism | -.10** | .04 | -.07 | .03 |
| Narcissism | -.11** | -.01 | .05 | .00 |
| Psychopathy | .02 | -.12** | -.05 | -.04 |
| Sadism | -.18** | -.04 | -.02 | .03 |
| Number of children | .40** | -.13** | .07 | -.17** |
| Age of first reproduction | -.02 | .33** | .15** | .17** |

Notes: * - *p* < .05; ** - *p* < .01
